# Identification of Components Associated with Thermal Acclimation of Photosystem II in *Synechocystis* sp. PCC6803

**DOI:** 10.1371/journal.pone.0010511

**Published:** 2010-05-06

**Authors:** John G. Rowland, Xin Pang, Iwane Suzuki, Norio Murata, William J. Simon, Antoni R. Slabas

**Affiliations:** 1 School of Biological and Biomedical Sciences, Durham University, Durham, United Kingdom; 2 Graduate School of Life and Environmental Sciences, University of Tsukuba, Tsukuba, Ibaraki, Japan; 3 Department of Regulation Biology, National Institute for Basic Biology, Okazaki, Aichi, Japan; Newcastle University, United Kingdom

## Abstract

**Background:**

Photosystem II (PSII) is the most thermally sensitive component of photosynthesis. Thermal acclimation of this complex activity is likely to be critically important to the ability of photosynthetic organisms to tolerate temperature changes in the environment.

**Methodology/Findings:**

We have analysed gene expression using whole-genome microarrays and monitored alterations in physiology during acclimation of PSII to elevated growth temperature in *Synechocystis* sp. PCC 6803. PSII acclimation is complete within 480 minutes of exposure to elevated temperature and is associated with a highly dynamic transcriptional response. 176 genes were identified and classified into seven distinct response profile groups. Response profiles suggest the existence of an early transient phase and a sustained phase to the acclimation response. The early phase was characterised by induction of general stress response genes, including heat shock proteins, which are likely to influence PSII thermal stability. The sustained phase consisted of acclimation-specific alterations that are involved in other cellular processes. Sustained responses included genes involved in phycobillisome structure and modification, photosynthesis, respiration, lipid metabolism and motility. Approximately 60% of genes with sustained altered expression levels have no known function. The potential role of differentially expressed genes in thermotolerance and acclimation is discussed. We have characterised the acclimation physiology of selected gene ‘knockouts’ to elucidate possible gene function in the response.

**Conclusions/Significance:**

All mutants show lower PSII rates under normal growth conditions. Basal PSII thermotolerance was affected by mutations in *clpB1*, *cpcC2*, *hspA*, *htpG* and *slr1674*. Final PSII thermotolerance was affected by mutations in *cpcC2*, *hik34*, *hspA* and *hypA1*, suggesting that these gene products play roles in long-term thermal acclimation of PSII.

## Introduction

Plants and micro-organisms must continually adapt to environmental changes. Such changes can be rapid or gradual dependent on the environmental niche of the organism and the challenge that is presented. The photosynthetic cyanobacterium *Synechocystis sp*. PCC6803 (hereafter referred to as *Synechocystis*) has been extensively used for investigations concerning signal perception and down-stream responses that occur following various environmental stresses. These have resulted in the elucidation of histidine kinases genes and response regulatory pathways involved with specific abiotic stresses and the generation of a *Δhik34* mutant with increased thermotolerance [1]. Studies on micro-organisms have increased our understanding of basic response pathways, some of which, such as the heat shock response, are conserved throughout nature. The origin of the higher plant chloroplast is believed to be the result of endosymbiosis of a cyanobacterial progenitor [2]. Studies on modern-day cyanobacteria have increased our understanding of photosynthesis.

The ability of an organism to withstand a particular stress and survive is dependent on both the strength and duration of the stress and the growth condition prior to the stress being applied. *Synechocystis*, like yeast and *Arabidopsis thaliana*, is thermally sensitive. Sudden exposure to high temperatures is frequently lethal. Exposure to an intermediate temperature prior to challenging with a normally lethal one can protect the organism in a process known as acclimation. In *Synechocystis*, acclimation phenomena are known to occur for a variety of stresses [3], [4], [5], [6], .

We are particularly interested in heat acclimation in *Synechocystis*. This organism offers an excellent experimental system in which to investigate this using both transcriptomic and proteomic approaches and is amenable to targeted gene deletion by homologous recombination [9].

The photosynthetic machinery located on thylakoid membranes in both cyanobacteria and higher plants is highly sensitive to heat inactivation, with photosystem (PS) II being the most sensitive component [10], [11], [12], [13].

The level of unsaturation of lipids in plants and cyanobacteria is also strongly influenced by temperature with higher levels of unsaturation seen at lower temperatures (see [14] and reviewed in [15]). Results from the analysis of lipid desaturase mutants in *Synechocystis* indicate that, within the normal level of unsaturation seen physiologically, lipid desaturation is not important in acclimation of the photosynthetic machinery [16], [17]. Studies in cyanobacteria have also shown that heat shock proteins (HSPs) can re-locate during thermal challenge and that binding of these proteins to membranes can alter both membrane stability, via upshift of the critical temperature for non-bilayer phase formation, and membrane fluidity [18], [19], [20], [21], which is potentially an additional mechanism by which cyanobacteria can maintain membrane integrity under stress conditions.

Protein stability and folding may play crucial roles in acclimation in a range of organisms. Much of the work in this area has been focused on HSPs and HSP104 homologs in particular. Whilst investigations have been performed to define the role of these HSPs in modulating cellular viability in response to heat stress, few studies report directly on the effects of heat acclimation on PSII activity. The involvement of proteins in PSII acclimation has been studied in both cyanobacteria and higher plants using both intact cells and a reconstitution approach [22], [23], [24]. These studies demonstrated that protein factors are critical to acquisition of thermotolerance. Furthermore, it has been found that thermal acclimation of PSII is dependent on *de novo* protein synthesis in *Chlamydomonas reinhardtii* [25]. Other as yet unknown proteins are also involved in thermal acclimation of PSII and it is far from clear how each component is involved in the process. In *Synechococcus*, ClpB is involved in acclimation of both PSII and survival [26] and a small HSP, *hspA*, has been shown to play a protective role for PSII activity during heat stress [27].

In the current study we have investigated the physiology and global transcriptional events associated with thermal acclimation of PSII in *Synechocystis*. We report on the detailed responses of gene transcripts, their overall response profiles and the generation and characterisation of mutants in early responsive genes that are involved in PSII acclimation.

## Results and Discussion

### Growth temperature influences PSII thermotolerance of *Synechocystis*

**Figure 1:**
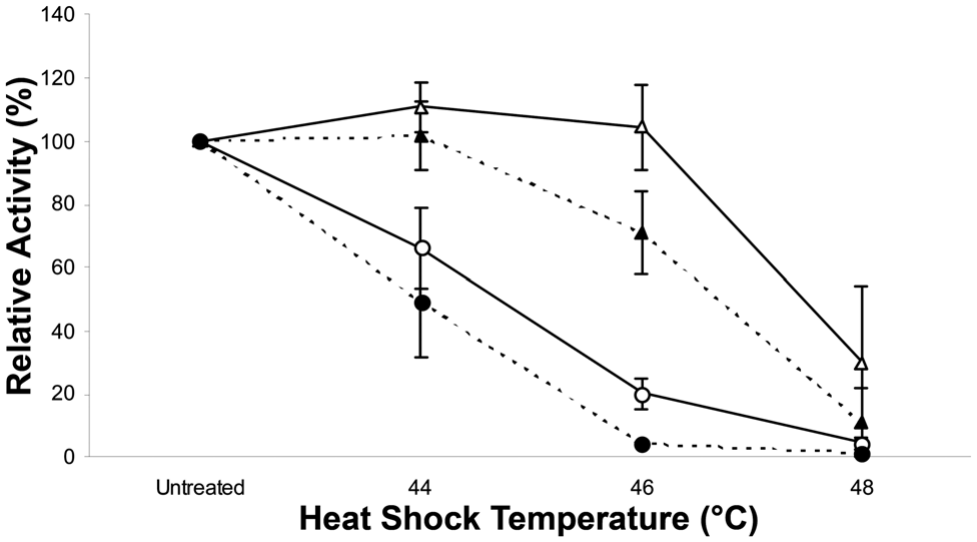
Effect of growth temperature on PSII thermotolerance. Triplicate *Synechocystis* cultures were grown at 25°C (circles) or 38°C (triangles) to A_730_ = 1. Aliquots were then taken and exposed to the growth temperature (Untreated control), 44°C, 46°C or 48°C for 1 hour (solid line) or 2 hours (dashed line) in the dark. PSII activity was measured in triplicate using a Clarke-type electrode. Data expressed as a percentage of the control PSII activity remaining following thermal challenge (*n* = 3).

In order to ascertain optimal conditions for measuring differences in PSII activity and to identify the degree to which PSII activity can be acclimated, we assessed the thermal stability of PSII from wild-type *Synechocystis* cultures grown at 25°C and 38°C and the effect of exposure to various temperatures for 1 or 2 hours (Fig. 1).

Growth temperature has a marked influence on PSII activity in *Synechocystis*, with thermotolerance being enhanced as a result of growth at higher temperatures. Of the conditions tested, cultures grown at 38°C and exposed to temperature treatments for 1 hour (open triangles, solid line) exhibited the greatest loss of PSII activity at 48°C. Incubation at 44°C or 46°C resulted in minimal loss of activity. Greater degrees of inactivation with 38°C cultures were observed following incubation periods of 2 hours (closed triangles, dashed line). Incubation at 46°C resulted in a reduction to 75% and no change was detected following a 44°C treatment.

Cultures grown at 25°C exhibited much greater susceptibilities to thermal inactivation of PSII. A 1 hour incubation period (open circles, solid line), reduced PSII activity to approximately 70% following 44°C treatment, 20% following 46°C treatment and exposure to 48°C resulted in near-total loss. Increasing incubation time to 2 hours (closed circles, dashed line) resulted in further inactivation of PSII, with a reduction in activity to approximately 50% and 5% resulting from incubation at 44°C and 46°C respectively.

From these studies it is clear that cultures grown at 38°C demonstrate enhanced PSII thermotolerance in comparison to those grown at 25°C. The greatest discrimination that can be reproducibly observed between the two cultures is provided using a heat treatment of 46°C and incubation period of 1 hour.

### Acclimation to elevated temperature enhances PSII thermotolerance

**Figure 2:**
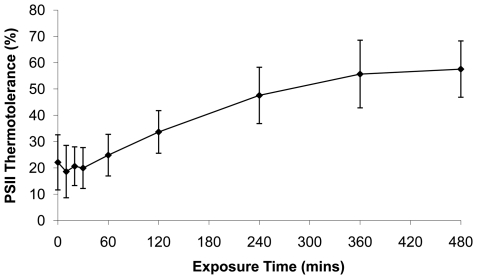
Acclimation of the photosynthetic machinery occurs within 480 minutes. Triplicate *Synechocystis* cultures were grown at 25°C to A_730_ = 1. The cultures were then shifted to 38°C and sampled over a time-course of 0 to 480 mins. Samples were treated for 1 hour in the dark at the growth temperature or at 46°C (thermal challenge). PSII activity in these samples was assessed in triplicate using a Clarke-type electrode. Data expressed as a percentage of the control PSII activity remaining following thermal challenge (*n* = 3).

Using our optimised assay conditions, we proceeded to establish the dynamics of the PSII acclimation response of wild-type *Synechocystis* when it is shifted from 25°C to 38°C for a period of up to 480 minutes (Fig. 2). The thermotolerance of PSII, expressed as a percentage of PSII activity remaining after thermal challenge, was initially low (approximately 20%) and remained at this level for the first 30 minutes to 1 hour following transfer to 38°C. This indicates that PSII is, unsurprisingly, initially very sensitive to thermal challenge. PSII thermotolerance then steadily increased over the remainder of the time-course, appearing to reach a plateau after 360–480 minutes of between 50 and 70% of the activity in non-challenged controls. This plateau suggests that the acclimation response is complete within this time period and that the cell has achieved a fully acclimated steady-state.

Acclimation of PSII thermotolerance thus occurs over a relatively long timeframe and includes an apparent lag phase, indicating that major alterations to the cellular metabolic machinery are required, but is essentially complete within 480 minutes.

### Acclimation induces a highly dynamic transcriptional response and reveals functional coexpression

Whole-genome cDNA microarray technology was used to monitor the kinetics and dynamics of the transcriptional response during acclimation of PSII. Samples were taken at 0, 10, 20, 30, 60, 120, 240 and 480 minutes after transfer of cultures from 25°C to 38°C. Using four biological replicates, we have identified all of the major transcriptional alterations that occur during acclimation using a 2-fold change cut-off threshold.

The complete dataset is available in Supporting Information, Table S1. In the complete dataset, thermal acclimation of wild-type cultures resulted in the altered expression of 176 genes, with 101 being induced and 75 repressed. A large number of these genes were annotated as hypothetical proteins (70 genes) and ORFs relating to ribosomal components (25 genes). Only 81 genes in the dataset were annotated in the genome database. Alterations in transcript abundance were highly dynamic, with many transcripts exhibiting large fold-changes and the largest changes occur very early in the time-course.

Between 240–480 minutes of acclimation the majority of responsive genes demonstrated a minimal rate of change, where there is little difference in expression between the penultimate and final time-points, suggesting that the transcriptional response was essentially complete.

Evaluation of the validity of the microarray data was performed by triplicate qRT-PCR analysis of selected transcripts (Table 1). Overall, a good correlation between both methods was found. Comparison of the array data with qRT-PCR derived data for expression level alterations between the 0 and 10 minute samples resulted in an R-squared correlation coefficient of 0.8. This increased to 0.95 when comparing the expression changes between the 0 and 20 minute samples.

**Table 1:** Verification of microarray data by qRT-PCR.

Gene Name	Microarray						qRT-PCR					
	*0 vs. 10 min*			*0 vs. 20 min*			*0 vs. 10 min*			*0 vs. 20 min*		
*hspA*	12.08	±	1.08	38.47	±	6.13	15.04	±	9.65	34.87	±	16.86
*clpB1*	15.70	±	0.75	27.66	±	3.20	10.79	±	2.70	17.33	±	3.75
*slr1674*	18.55	±	1.57	16.78	±	0.43	15.57	±	6.34	14.21	±	8.13
*groEL-1*	4.42	±	0.48	6.26	±	2.15	3.65	±	0.81	7.39	±	2.96
*sigB*	6.50	±	0.15	6.50	±	0.01	4.73	±	1.41	6.44	±	2.68
*htpG*	18.84	±	1.38	16.26	±	0.33	9.06	±	4.73	16.67	±	2.15
*psaD*	2.71	±	0.09	3.01	±	0.15	1.28	±	0.04	1.40	±	0.24
*rplJ*	0.92	±	0.03	0.86	±	0.02	0.93	±	0.06	1.09	±	0.16
*desA*	0.22	±	0.00	0.31	±	0.01	0.18	±	0.09	0.26	±	0.11
*desD*	0.21	±	0.01	0.21	±	0.02	0.13	±	0.01	0.18	±	0.01

Quantitative real-time PCR was performed in triplicate for selected ORFs (indicated), and the data compared to that derived from microarray analysis by calculating R-squared correlation coefficients (0 vs. 10 mins data: r^2^ = 0.8; 0 vs. 20 mins data: r^2^ = 0.95).

**Figure 3:**
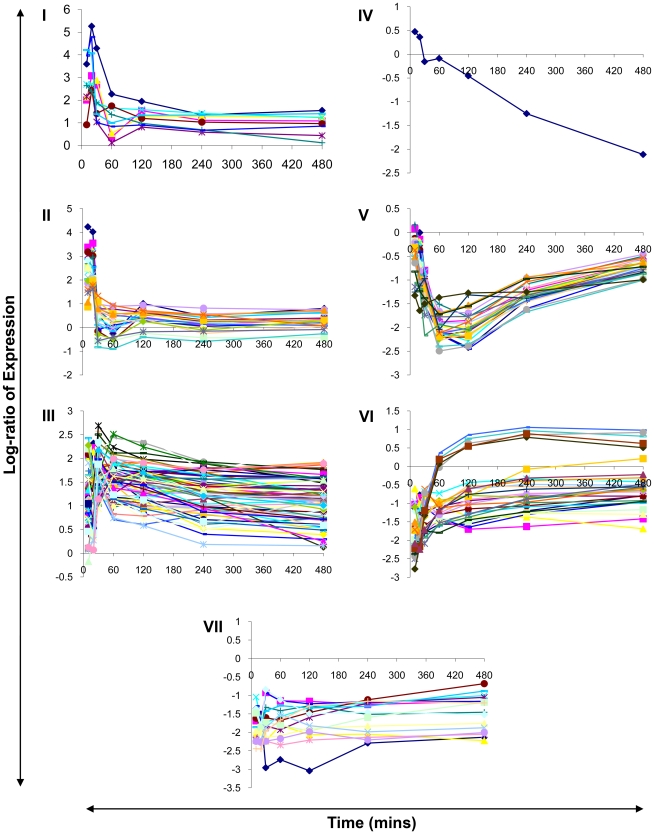
Microarray analysis reveals highly dynamic transcriptional responses. The data for significantly differentially expressed ORFs were clustered into 7 groups. **I**: Transient induction but continued to 480 mins; **II**: Transient induction in 20 mins, then decreased to initial level; **III**: Immediate induction and continued to 480 mins; **IV**: Gradual repression to 480 mins; **V**: Repressed followed by gradual recovery; **VI**: Transient repression followed by recovery; **VII**: Repressed and continued to 480 mins. The log-ratio of differential expression is presented on the Y-axis with time denoted in minutes on the X-axis.

The expression of all 176 responding genes were classified by k-means clustering (see [28] for a review of clustering approaches) into 7 distinct response profiles (Fig. 3). The resulting groupings suggest the existence and operation of common regulatory influences or a requirement for co-expression as, in many cases, genes with similar or related functions are present in the same group. The general characteristics of these groups are discussed below.

For example, genes in groups I and II, both induced early in the response, primarily encode stress-related proteins and proteins with known functions as chaperones. In group V, characterised by immediate repression followed by gradual recovery, almost all genes encode components of the ribosomal 50S and 30S subunits. The remaining groups (III, IV, VI, and VII) of genes also contain functionally related genes but the groups as a whole are not characterised by any one function. This may suggest a requirement for coordinated expression of multiple functions for successful acclimation to elevated temperature.

Based on transcript response profiles, only members of groups I, III, IV and VII remained differentially expressed compared to control at the end of the acclimation time-period. A considerable number of these acclimation-responsive transcripts are annotated as hypothetical proteins highlighting the fact that many of the molecular components and mechanisms involved during thermal acclimation are largely unknown.

The highly dynamic nature of the responses suggests that the organism is undergoing substantial modification of its cellular machinery and associated metabolism and that the transcript response profiles reflect the cascade of events required to achieve steady-state. The majority of transcriptional alterations occur almost immediately upon transfer to elevated temperature. Some of these responses are short-lived and return to control levels within 60 minutes, while other responses are sustained throughout the acclimation period. The acclimation response appears to be composed of two overlapping phases; a transient phase and a sustained phase. Transient transcript alterations may be essential players in the ability of the cell to progress to a thermotolerant phenotype. Conversely, sustained phase transcripts may contribute directly to thermotolerance itself or simply reflect metabolic requirements under the new condition. The potential contribution of responsive genes in thermal acclimation is discussed below in the context of major functional processes that they participate in.

#### Heat Shock & Other Stress Responses

The expression of HSPs is highly induced within 10–20 minutes of acclimation and this may represent a general stress response, maintaining appropriate protein structure and function of essential cellular machinery at higher growth temperatures via their chaperonin/protease activities [29], [30], [31]. After 30 minutes of acclimation, a divergent HSP expression profile is apparent, with those in Group II (*htpG*, *dnaJ* & *dnaK2*) returning to control levels and those in Group I (*hspA*, *clpB1*, *groES*, *groEL1* & *groEL2*) remaining at a relatively elevated level. Group II HSPs may be required only at the onset of the response, with specific functions or roles distinct from HSPs in Group I. Group I HSPs may be required primarily for survival of the original heat-shock but also for maintaining the fully acclimated phenotype of the organism during sustained elevated temperatures. Indeed, homologs of ClpB1 have been identified as being essential for cellular thermotolerance in yeast [32] and plants [33], [34]. ClpB has also been reported as involved in thermotolerance in *Synechococcus* sp. PCC7002 [26]. HspA has been reported to be inserted into membranes upon heat shock resulting in membrane stabilisation [20] and may play a role in the immediate stabilisation of membranes until membrane lipids can be modified via the action of desaturases.

In addition to the heat shock response, protection from reactive oxygen species (ROS) is implied by elevation of water-soluble carotenoid and the iron-dependent SodB transcripts [35], [36]. The co-expression of *futA1*, involved in iron transport [37], [38], may be functionally linked to SodB activity. Detoxification of ROS may protect PSII as, in spinach, ROS-mediated damage of the D1 protein may be the primary cause for release of manganese and the PsbO protein from PSII [39]. Additionally, FutA1 is localised predominantly intracellularly on the cytoplasmic side of the thylakoid membranes and also co-purifies with PSII [40]. It has been reported that FutA1 and homologues in other cyanobacteria play a role in protecting the acceptor side of PSII from damage under conditions of iron-limitation and peroxide treatment [40], [41]. It has been speculated that such protection could be mediated either directly or by improving iron availability to the D1/D2 reaction centre (reviewed in [42]). The PSII repair cycle, involving turnover of D1/D2, is more active at higher growth temperatures and therefore a greater requirement for iron exists. This second function for FutA1 may be more physiologically relevant to thermal acclimation of PSII as our study has not identified differential expression of any of the other components of the iron uptake system.

It seems likely that the regulation of expression of these HSPs and ROS-related transcripts may be under the control of both the SigB transcription factor and the histidine kinase, Hik34, both of which have been reported to be involved in the regulation of the heat shock response [1], [43]. SigB has been reported to be involved in short-term heat shock and required for acclimation [44], although the *ΔsigB* mutant remained capable of increasing its thermotolerance via acclimation. This suggests that non-SigB mediated mechanisms are also involved in thermal acclimation. Our data on the expression of *sigB* and *hik34* are consistent with a gene regulatory model including the sigma factors and *hik34* that has been proposed by others [43]. It is possible that SigB and Hik34 function cooperatively to generate the apparent divergent regulation of HSPs and other stress response proteins discussed above, with a subset of these being specifically maintained at elevated levels.

#### Photosynthesis & Associated Components

Genes encoding structural components of the phycobillisome *cpcC1*, *cpcC2* and *cpcD* are elevated and maintained at relatively higher levels. Although these are present in the genome in an operon with phycocyanin-encoding *cpcA* and *cpcB*, the phycocyanin ORFs do not exhibit the same response, suggesting that the linker proteins are less stable at higher temperatures than the phycocyanins. In contrast, *cpcG2* is highly down-regulated. Phycobillisomes containing CpcG2 have been shown to lack the phycocyanin core and preferentially transfer energy to PSI [45]. The altered abundance of phycobillisome components suggests remodelling of the phycobillisome complexes and is supported by the elevated levels of *nblA1* and *nblA2* also observed. Degradation of phycobillisomes by NblA has been reported under conditions of nutrient-limitation and high-light and may serve as a nitrogen-source and/or photoprotective mechanism by reducing the amount of light arriving at the reaction centres [46], [47]. Additionally, the increased expression of the *cpc* genes may allow optimisation of directional energy transfer from phycocyanin into the photosystems (reviewed in [48]).

Interestingly, only one transcript related to each of the photosystems was elevated in fully acclimated cultures as detected by microarray analysis. This was the *psaD* transcript, encoding a subunit of the reaction centre complex in PSI, and *ctpA,* responsible for the processing of D1 protein component of the reaction centre complex of PSII into its catalytically active form. This implies possibly elevated turnover/repair of PSII, although proof of this would require further experimentation. Increased levels of *psaD* could not be unequivocally confirmed by qRT-PCR analysis (Table 1). It should also be noted that the expression of PSI genes is generally highly coordinated and so the differential expression of *psaD* in isolation would be unexpected.

#### Lipid Metabolism & Lipid Desaturases

All known fatty-acid desaturases (*desA*, *desB*, *desC*, *desD*) are repressed in the first 20 minutes of acclimation. The expression of *birA* is elevated. The protein encoded by *birA* is responsible for attachment of biotin to the biotin-dependent acetyl-CoA carboxylase (ACC) [49]. ACC is responsible for the generation of malonyl-CoA required for *de novo* fatty acid synthesis. Elevation of *birA* and repression of the desaturases suggests that fatty acid synthesis may be elevated and that *de novo* synthesised lipids remain saturated as the desaturase activity could be low. This could result in altered lipid composition of cellular membranes.

Membranes contain a high proportion of unsaturated fatty acids. The biosynthesis of polyunsaturated fatty acids involves stepwise desaturation via acyl-lipid desaturases, with desaturation by DesC at the Δ9 position, producing 18∶1 fatty acids, occurring first. Interestingly, we have observed a tightly controlled initial down-regulation of *desC*, followed by an immediate return to original levels. The stability of *desC* mRNA has been shown to be unaffected by temperature in *Synechococcus* sp. PCC7002 and *Synechocystis* [50], [51]. To the best of our knowledge, this is the first report of temperature-responsive modulation of the expression of *desC*. Rapid down-regulation of *desC* would result in immediate removal of substrates for the other desaturase enzymes and could effectively switch off polyunsaturated membrane lipid biosynthesis. It is well known that at higher temperatures the level of polyunsaturated fatty acids in membranes decreases, with 18∶2/18∶3 fatty acids being particularly affected. Perhaps the initial drop in DesC activity acts as an additional brake on 18∶2/18∶3 biosynthesis and, once the level of activity of other desaturases has been reduced, the reappearance of DesC would allow both saturated and 18∶1 unsaturated fatty acids to be synthesised for insertion into membranes. The repression of desaturases therefore maintains membrane integrity and the correct functioning of membrane associated processes such as photosynthesis. Increased membrane integrity has been linked to temperature sensitivity in both plants [52], [53], [54], [55], [56] and cyanobacteria [57].

#### Other Processes

Transcripts encoding ribosomal subunits are down-regulated, a feature common to many stress responses. Ribosomal transcript expression resumes after 60 minutes, suggesting that the initial stress response has completed and “normal” protein translation can resume. This coincides with the rapid return to original levels of a functionally diverse group of genes (group VI), indicating the re-establishment of metabolic processes that were inhibited during the initial mitigation of the stress.

### Selection and generation of mutant strains

To study the importance of genes involved in the early-response to thermal acclimation of *Synechocystis*, mutants affected in genes encoding ClpB1, HspA, HypA1, CpcC2, HtpG and Slr1674 were generated by *in vitro* transposon mutagenesis. These Group I (*clpB1*, *hspA*, *cpcC2*, *slr1674*) and Group II (*htpG*, *hypA1*, *hik34*) genes were rapidly induced upon transfer to the acclimation temperature and may play important roles in initial events involved in thermal acclimation of PSII. *Synechocystis* was subsequently transformed with plasmids containing inactivated genes for integration into the genome by homologous recombination. All transformants were fully segregated prior to further analysis (Supporting Information, Figure S1). We chose to generate our own mutant strains using the same parental wild-type as there may be differences unrelated to the mutation between strains obtained from other labs or a culture collection.

### Only strain *ΔhtpG* is significantly impaired in growth following transfer from 25°C to 38°C

**Figure 4:**
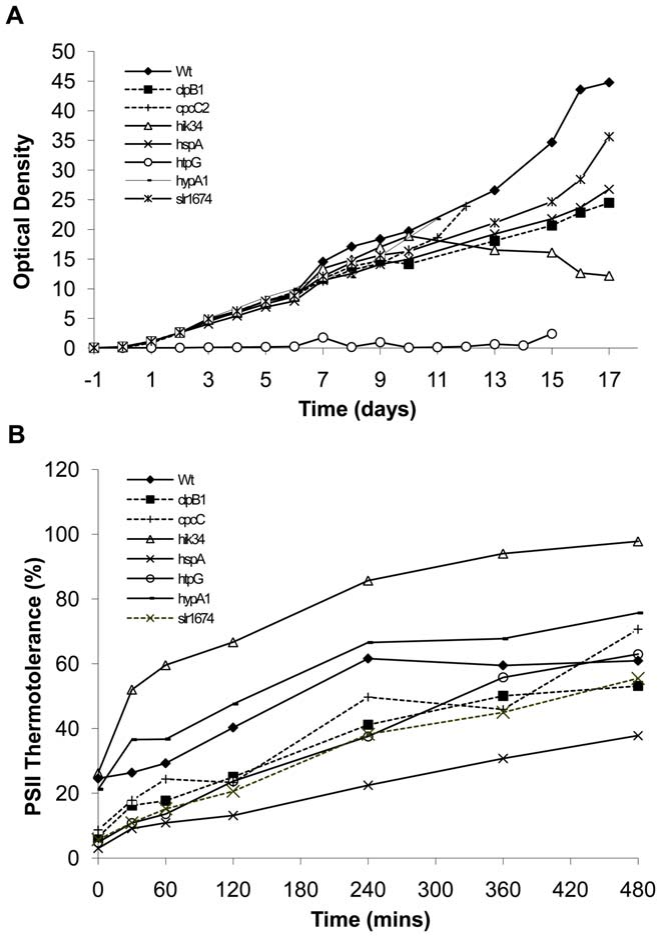
Characterisation of the acclimation response of knock-out mutants. **A**. The growth of *Synechocystis* mutant cultures during acclimation was assessed in triplicate by monitoring changes in optical density (A_730_). Cultures were inoculated at an OD of 0.05 and grown at 25°C for 1 day. Cultures were transferred to 38°C and sampled daily for up to 17 days (*n* = 3). **B**. Triplicate *Synechocystis* mutant cultures were grown at 25°C to A_730_ = 1. The cultures were transferred to 38°C and sampled over a time-course of 0 to 480 minutes. Samples were then treated for 1 hour in the dark at the growth temperature or at 46°C. PSII activity in these samples was assessed in triplicate using a Clarke-type electrode. Data are expressed as the fold-change in PSII thermotolerance (*n* = 3).

Analysis of growth for all strains under acclimation conditions revealed that none of the mutants were significantly different from wild-type over the acclimation period of 480 minutes that was used to profile PSII physiology, although differences are apparent after an extended acclimation period. Changes in cell culture density for all strains over an acclimation period of many days are shown in Figure 4A. Wild-type culture density increased over the entire period. Alterations in density for strains *ΔcpcC2* and *ΔhypA1* were not significantly different from wild-type. Notably, the *ΔhtpG* mutant strain (open circles) largely failed to increase in density over the same period, but remained viable. All other strains tested demonstrated reduced growth compared to wild-type over the acclimation period, but this difference only became significant after a period of at least 7 days. The response of the *Δhik34* mutant (open triangles) is notable in that after approximately 10 days of growth at the acclimation temperature its density progressively decreased. As there was no significant difference in strain growth within the first 480 minutes, meaningful analysis of PSII acclimation can be performed in this time window that will not be affected by differences in growth rates.

### Comparative physiology of wild-type and mutant strains reveals complex roles for selected genes in thermal acclimation of PSII

Having established that there were no differences in growth rates between wild-type and mutant strains during the 480 minute acclimation period from 25°C to 38°C, PSII activity and thermotolerance under conditions of thermal acclimation were examined. Numeric data for absolute rate measurements are presented in Tables 2 and 3. These data are also expressed as percentage thermotolerances in Table 4 and shown visually in Figure 4B. Interpretation of acclimation-induced changes in PSII activity pre- and post- 46°C heat treatment requires a detailed consideration of the effects of the mutations on **[a]** the basal rate of PSII activity (i.e. at the 0 time-point), **[b]** differences in PSII activity resulting from the temperature shift, **[c]** basal PSII thermotolerance and **[d]** final PSII thermotolerance achieved after transfer from 25°C to 38°C while allowing for any differences in PSII activity that may be caused by the temperature shift.

**Table 2:** Absolute rates of PSII activity during acclimation of *Synechocystis* strains.

Time (mins)	Wt	clpB1	cpcC2	hik34	hspA	htpG	hypA1	slr1674
0	745.2±14.9	636.5±18.5	594.8±56.3	480.5±8.6	645.9±15.0	611.7±16.4	490.9±17.0	586.9±16.5
30	668.7±21.3	545.8±15.4	529.0±61.5	398.0±10.0	583.8±15.5	584.4±16.9	462.4±24.2	521.4±15.8
60	636.5±13.7	503.2±13.0	484.2±42.5	394.0±15.1	520.1±10.9	574.5±9.8	459.5±16.8	566.1±9.9
120	594.2±20.1	469.1±22.8	447.0±54.8	386.3±14.2	575.1±21.6	541.1±14.9	435.0±10.9	542.8±14.6
240	521.3±20.1	352.6±13.9	365.3±78.6	342.6±20.3	491.6±15.3	401.6±26.3	374.4±16.7	459.6±13.7
360	452.1±19.3	294.4±7.0	384.4±77.6	324.3±6.6	430.3±18.7	362.3±23.1	382.5±18.7	404.5±13.9
480	409.1±11.5	278.6±12.9	419.9±57.3	315.4±4.4	364.5±11.7	325.7±16.4	333.3±13.9	346.6±13.5

Absolute PSII rates of wild-type and selected mutant cultures that had been acclimated from 25°C to 38°C over time. Data are shown as the mean±1 standard deviation of three independent replicate experiments and expressed as µmol O_2_ mg^−1^ Chlorophyll h^−1^.

**Table 3:** Residual rates of PSII activity during acclimation of *Synechocystis* strains.

Time (mins)	Wt	clpB1	cpcC2	hik34	hspA	htpG	hypA1	slr1674
0	183.2±24.2	41.5±6.4	51.6±6.5	126.0±1.9	19.4±4.1	29.9±5.3	104.3±4.1	33.4±6.7
30	176.3±39.5	88.8±6.2	94.5±6.3	207.1±8.8	53.2±6.3	63.1±10.3	169.2±6.3	56.9±7.9
60	186.3±55.2	89.1±6.9	118.0±5.5	234.8±3.9	56.5±6.7	78.2±13.7	168.8±6.7	86.2±10.7
120	239.5±46.7	117.7±6.0	104.4±6.5	257.6±5.1	75.6±8.9	128.3±12.6	207.0±8.9	112.0±12.0
240	321.1±64.0	145.2±9.1	181.6±25.8	293.7±6.3	110.6±14.5	150.8±12.9	249.2±14.5	175.7±14.2
360	268.9±79.8	147.4±8.9	176.4±21.5	305.0±9.7	132.4±10.4	202.2±13.0	259.1±10.4	182.1±14.1
480	249.2±59.4	148.1±10.1	296.7±60.4	308.6±6.9	137.8±10.5	205.1±13.4	252.3±10.5	192.3±13.6

Residual PSII rates after 46°C treatment of wild-type and selected mutant cultures that had been acclimated from 25°C to 38°C over time. Data are shown as the mean±1 standard deviation of three independent replicate experiments and expressed as µmol O_2_ mg^−1^ Chlorophyll h^−1^.

**Table 4:** Progression of PSII thermotolerance during acclimation of *Synechocystis* strains.

	Wt	clpB1	cpcC2	hik34	hspA	htpG	hypA1	slr1674
Basal Thermotolerance	24.6	6.5	8.7	26.2	3.0	4.9	21.2	5.7
Final Thermotolerance	60.9	53.2	70.7	97.8	37.8	63.0	75.7	55.5
Basal % of Wt	100	26.4	35.4	106.5	12.2	19.9	86.2	23.2
Final % of Wt	100	87.4	116.1	160.6	62.1	103.4	124.3	91.1
Basal Susceptibility	NA	−	−	•	−	−	•	−
Final Susceptibility	NA	•	+	+	−	•	+	•

Acclimating cultures (25°C transferred to 38°C) were sampled over the acclimation period and PSII activity measured with and without 46°C heat treatment. Thermotolerance was then calculated as the percentage of control PSII activity that remained in cultures following the 46°C heat treatment. “Basal % of Wt” is the basal thermotolerance of the mutant compared with wild type and is expressed as a percentage. “Final % of Wt” is the final thermotolerance of the mutant compared with wild type and is expressed as a percentage. “Susceptibility” is a qualitative assessment of whether PSII activity is more or less susceptible to thermal inactivation compared with the wild type.+: Tolerant, −: Susceptible, •: No difference, NA: Not applicable.

For clarity, we define the terms we employ in this manuscript to describe PSII physiology. *Residual activity* is the absolute oxygen-evolution rate that PSII is capable of after heat treatment at 46°C for 1 hour. *Thermotolerance* is a relative measure of the amount of activity that is retained after 46°C heat treatment, expressed as a percentage of the control PSII activity at that time point for that strain. *Basal thermotolerance* refers to that measured at the start of the time-course. *Final thermotolerance* refers to the thermotolerance that is achieved by each strain at the end of the time-course.

#### Basal PSII activity is impaired in all mutant strains and is progressively lowered following transfer to the acclimation temperature

We assayed the PSII activity of wild-type and mutant strains following transfer from 25°C to 38°C, using cultures that had been fully conditioned to 25°C over several weeks prior to the experiments to ensure full acclimation. All mutants had a lower PSII activity than wild-type at the beginning of the time-course (zero time-point, Table 2). This suggests that mutation of these genes results in a decrease of PSII activity and that the genes are likely to be involved in the maintenance of higher activity in the wild-type. Wild-type cultures exhibited a gradual decline in PSII rates over the acclimation period, with activity after 480 minutes approaching half the initial rate. Similarly in all the mutant strains, PSII activity gradually decreased over the same time period. The gradual decline in PSII activity during acclimation of all strains suggests the existence of processes that modulate PSII activity in response to changes in growth temperature. This is the first demonstration of long term effects of mutations affecting PSII activity as opposed to effects directly following heat shock.

#### All strains tested are capable of at least partial improvement of residual PSII activity during acclimation

Examination of PSII activity following thermal challenge at 46°C for 1 hour demonstrates that the residual PSII activity in wild-type and all mutant strains increases during acclimation to 38°C over a 480 minute period (Table 3). In all mutants, the residual PSII activity at 0 minutes was less than that of the wild-type. Strains *ΔclpB1* and *ΔhspA* demonstrated residual PSII activities after 480 minutes that were substantially lower than wild-type. The remaining mutant strains had residual PSII activities after 480 minutes that were similar to wild-type.

#### Most mutants are severely impaired in basal PSII thermotolerance but this is not predictive of final PSII thermotolerance after acclimation

For our mutants, with the exception of *Δhik34* and *ΔhypA1*, the basal thermotolerance is severely compromised compared with the wild-type (Table 4 and Figure 4B), consistent with these genes playing important roles in protection from heat-shock.

Final thermotolerance at the end of the acclimation period increased substantially for wild-type and all mutant strains in comparison to their basal thermotolerance. Strains *ΔclpB1*, *ΔhspA* and *Δslr1674* exhibited thermotolerances that were less than that achieved by the wild-type (Table 4 and Figure 4B). All other mutants exhibited PSII thermotolerances that were equal to or greater than wild-type. There does not appear to be any strict correlation between basal thermotolerance and that which can be achieved after acclimation.

This indicates that, with the exception of Hik34 and HypA1, the products of the other genes that we have mutated are important for the immediate protection of PSII from heat. We have previously shown that the *Δhik34* strain has elevated expression of HSPs that can be further induced by heat [1]. This explains why this strain does not exhibit a severely impaired basal thermotolerance phenotype and is able to achieve a similar or greater final thermotolerance than the wild-type. This “upshift” in PSII thermotolerance is more pronounced after long-term thermal acclimation, possibly due to additive effects of other adaptive mechanisms.

HypA1 is involved in nickel insertion into the bidirectional hydrogenase active site and is essential for hydrogenase activity in *Synechocystis* [58]. The precise function of the hydrogenase has been a matter of some debate but has been postulated to act during fermentation [59], [60], as a valve for low-potential electrons generated during photosynthesis under stress conditions [61], [62] and to also form a part of respiratory complex I [63]. In the context of this study, the hydrogenase is probably acting to regulate the flow of electrons through the photosynthetic electron transport chain. Precisely why inactivation of the hydrogenase, caused by deletion of *hypA1*, would result in increased PSII thermotolerance is unclear but probably involves alterations in the redox state of the plastoquinone pool. Altered abundance of photosynthesis components, including both photosystems and cytochrome b_6_/f, have been observed in a hydrogenase mutant [62] and it seems likely that these alterations may be contributing to improved PSII thermotolerance in the *ΔhypA1* strain. Further work is necessary to define the potential role of HypA1 in thermal acclimation of PSII and thermotolerance.

From the data presented in Table 4 and Figure 4B we can begin to differentiate between involvement in the classical heat shock response and acclimation. It is clear that ClpB1, CpcC2, HspA, HtpG and the gene product encoded by *slr1674* are all involved in protection of PSII from thermal damage caused by the 46°C heat treatment. Of these gene products, only HspA appears to be required for maintaining wild-type levels of final thermotolerance. HspA is therefore involved in both the classical short-term heat shock response and longer-term PSII acclimation. ClpB1, CpcC2, HtpG and the gene product encoded by *slr1674*, while clearly involved in the short-term heat shock response, do not appear to play major roles in longer-term acclimation of PSII. Interestingly, the absence of Hik34 and HypA1 appears to improve the longer-term acclimation of PSII while having minimal roles in the protection of PSII in the absence of acclimation. To summarise, it appears as though ClpB1, CpcC2, HtpG and the *slr1674* gene product are involved in only the heat shock response. Hik34 and HypA1 both affect the PSII acclimation process but appear to have minimal effects during the initial heat shock. HspA is involved in both the heat shock response and acclimation processes.

#### Measurement temperature in the oxygen electrode has little influence on the observable PSII thermotolerance

Defining the most appropriate temperature for taking measurements using the oxygen-electrode is problematic as at either the 0 or 480 minute time-point there is a disparity between the measurement temperature (25°C) and the growth temperature of the organism (25°C, acclimating to 38°C). Accordingly, we have measured PSII thermotolerance of the wild-type strain in an additional experiment, this time using a measurement temperature of 38°C. Data are provided in Supporting Information, Figure S2. Under these circumstances we do not see a decline in PSII activity upon transfer from 25°C to 38°C during the time-course. Importantly, the data on basal PSII thermotolerance compared with final thermotolerance closely mirrors that obtained when performing the measurements at 25°C, indicating that the measurement temperature does not affect the observed thermotolerance. In effect, acclimation as defined by an increase in thermotolerance is seen to occur to the same extent under both sets of conditions. The decline in PSII activity seen when measuring at 25°C is probably related to a cold-sensitivity phenomenon.

### Concluding Statement

We are primarily interested in identifying components that are involved in regulating the fundamental process of photosynthesis and the activity of PSII in particular. We have performed physiological studies on PSII thermotolerance and the influence of growth temperature that has allowed us to optimise conditions to investigate thermal acclimation of PSII in *Synechocystis*. We have found that complete acclimation of PSII requires 480 minutes after transfer to the new growth temperature. Whole-genome microarray studies over this time period have been performed and have identified seven distinct transcriptional response profiles, revealing a highly dynamic transcriptional response program. We are aware of only one other report on transcriptomic analysis of responses to thermal acclimation [64]. That extensive body of work was performed in *Arabidopsis thaliana* and used two different acclimation treatments. In common with our study, they identified induction of transcripts for HSPs and repression of transcripts for chloroplastic ribosomal proteins. However, that study did not investigate the association of the transcriptional responses with alterations in PSII activity and PSII thermotolerance.

We have investigated the effect of mutations in selected early responsive genes with respect to both basal PSII thermotolerance and final thermotolerance following acclimation. Surprisingly, mutations in all of the genes selected caused a decrease in the absolute basal rate of PSII activity. Additionally, all strains were capable of at least partial acclimation of PSII thermotolerance.

Basal PSII thermotolerance was affected by mutations in *clpB1*, *cpcC2*, *hspA*, *htpG* and *slr1674*. It is easy to understand how the three HSPs (*clpB1*, *hspA*, *htpG*) affect thermotolerance but the mechanism for a component of the phycobillisome (*cpcC2*) and a protein of unknown function (*slr1674*) is less clear. Further investigation of the *slr1674* gene product may identify it as a classical HSP. Final PSII thermotolerance was clearly affected by mutations in *cpcC2*, *hik34*, *hspA* and *hypA1*. This suggests that these transcripts and their products play roles in long-term thermal acclimation of PSII.

We have further defined the roles of ClpB1 and HtpG in acclimation as being limited to the classical heat shock response, with limited or no involvement in longer-term acclimation of PSII. This adds to the previous reports of involvement of the *Synechococcus* homolog, ClpB, in protection of PSII from short-term heat stress [26]. We have also now shown that HspA is involved in longer-term acclimation of PSII in addition to its previously reported involvement in short-term heat shock [27].

The precise nature of the roles of the selected genes and their products in PSII acclimation remains to be determined but probably involve pathways with many additional components. Their contribution to thermal acclimation of PSII is likely to be complex. Future transcriptomic and proteomic studies on the mutants may help to elucidate the pathways involved and define the roles of these genes in more detail.

## Materials and Methods

### Strains and Culture Conditions

Glucose-tolerant *Synechocystis* sp. PCC 6803, was routinely grown photoautotrophically at 34°C in BG-11 medium [65] under constant illumination at 70 µmol photons/m^2^/s and aeration with sterile air containing 1% CO_2_. For maintenance of mutants, 50 µg/ml kanamycin was included in the culture medium. For experiments requiring an initial growth temperature of 25°C, all cultures were transferred to 25°C and sub-cultured at this temperature over several weeks prior to the experiment to ensure complete acclimation.

### Generation and Selection of Mutants

Mutant strains were generated by transposon mutagenesis. The coding sequences and neighbouring sequences were amplified by PCR, using primers designed using the complete *Synechocystis* genome sequence [66]. The approximately 2 kb PCR products were cloned into pT7-Blue (Novagen/MerckBiosciences, www.merckbiosciences.com). A kanamycin resistance cassette was introduced into these plasmids by *in vitro* transposition using the EZ: Tn5 <Kan-2> insertion kit (Epicentre Biotechnologies, www.epibio.com). Plasmids in which an insertion was identified within the target gene and with flanks of at least 500bp of genomic sequence were selected for generation of mutant strains. Transformation of *Synechocystis* has been described previously [67]. Transformants were initially selected on BG-11 agar containing kanamycin at 10 µg/ml, whilst the segregation of clones was performed by restreaking of primary clones on plates supplemented with kanamycin at 50 µg/ml several times (at least three transfers).

### Experimental Replication and Statistical Analysis

Unless stated otherwise, all experiments were performed at least three times using independent biological replicates. Statistical evaluation of data employed the two-tailed t-test with an assumption of homoscedasticity (equal variance).

### Acclimation of *Synechocystis*

*Synechocystis* cultures were grown at 25°C in BG-11 medium, with light and gas supplied as for routine culture, to an optical density (A_730_) of approximately 1.0. Cultures were then transferred to 38°C without disrupting illumination or aeration. Culture aliquots were taken for physiological characterisation and RNA extraction at various time-points both before and after the transfer.

### Physiological Characterisation

#### Growth

Growth and cell density were monitored by the OD_730_ of cultures with or without dilution using a UV/Vis spectrophotometer (Ultrospec 1100 Pro, GE Healthcare, www.gehealthcare.com). Triplicate measurements were taken for each of three independent biological replicates.

#### Activity of PSII

PSII activity in cells before and after heat-shock was measured using a Clark-type oxygen electrode. All measurements were performed in triplicate per biological replicate. Three biological replicates were performed, preventing measurement inaccuracies from influencing the data. The durations and temperatures used for heat-shock are designated in the results and were always performed in the dark. Samples were equilibrated to 25°C prior to measurement. The PSII-mediated electron transport from water to benzoquinone (BQ) was measured at 25°C in BG-11 medium supplemented with 1 mM BQ and 1 mM K_3_Fe(CN)_6_. Red actinic light at an intensity of 640 W/m^2^ was provided by an incandescent lamp after passing through a heat-absorbing optical filter (HA50; Hoya, www.hoyafilter.com) and a red optical filter (R-60; Toshiba, www.toshiba.co.jp). Chlorophyll concentrations were determined as reported previously [68].

### Gene Expression Analysis

RNA samples were generated from three independent biological replicates. For each sample, a 50 ml aliquot of culture was mixed with an equal volume of ice-cold 5% phenol in ethanol to prevent cellular metabolic processes. Cells were centrifuged at 4,000 *g* for 5 min at 4°C and resuspended in an additional volume of fresh ice-cold 5% phenol in ethanol. After further centrifugation the pellets were immediately frozen and stored at −80°C prior to RNA extraction. Total RNA was extracted with hot phenol as described previously [69].

Microarray analysis was performed using a commercial *Synechocystis* cDNA microarray (CyanoCHIP, TaKaRa Bio Co. Ltd, www.takara-bio.com), covering 3,079 of the 3,264 open reading frames of the *Synechocystis* genome [66].

CyeDye-labelled cDNA was synthesised using a MMLV RNA fluorescent labelling kit (TaKaRa Bio). Array hybridization and washing were performed as described previously [6]. Images were acquired with a GMS418 array scanner (Affymetrix, www.affymetrix.com).

Quantification was performed using ImaGene V. 5.5 software (BioDiscovery, www.biodiscovery.com) as described previously [1]. Spot signal intensity was determined after local background subtraction and normalised based on total signal intensity of all genes on the array except for genes encoding rRNAs. This allows changes in specific transcript abundance to be calculated relative to total mRNA. Duplicated spots on the array allowed for evaluation of signals and exclusion of errors. Differential expression was defined as a two-fold change [8]. The data discussed in this publication have been deposited in NCBI's Gene Expression Omnibus [70] and are accessible through GEO Series accession number GSE21133 (http://www.ncbi.nlm.nih.gov/geo/query/acc.cgi?acc=GSE21133).

Quantitative real-time PCR was performed using SYBR Green I and data analysed as described previously[71]. This analysis was performed in triplicate using the same RNA samples as for microarray analysis. Oligonucleotide primer sequences for gene-specific amplification are provided in Supporting Information, Table S2.

## Supporting Information

Table S1 Transcriptional responses during acclimation to elevated temperature. Transcript responses during acclimation over a period of 480 mins were assessed using microarrays. Three independent replicates were used. Transcripts were then clustered into response profiles. I: Transient induction but continued to 480 mins; II: Transient induction in 20 mins, then decreased to initial level; III: Immediate induction and continued to 480 mins; IV: Gradual repression to 480 mins; V: Repressed followed by gradual recovery; VI: Transient repression followed by recovery; VII: Repressed and continued to 480 mins.
(0.12 MB XLS)

Table S2 Primer sequences for quantitative RT-PCR.
(0.03 MB XLS)

Figure S1 Confirmation of complete segregation of mutants. PCR was performed on wild-type (Wt) and potential mutant (Mut) genomic DNA using the indicated gene-specific primers. Expected amplicon sizes from Wt DNA were 1.6 to 1.8 kb. Complete segregation is indicated by the absence of a product in the Mut lanes at the Wt band size.
(6.18 MB TIF)

Figure S2 Acclimation of PSII measured at 38°C. Duplicate *Synechocystis* wild-type cultures were grown at 25°C to A_730_ = 1. The cultures were then shifted to 38°C and sampled over a time-course of 0 to 480 minutes. Samples were then treated for 1 hour in the dark at the growth temperature or at 46°C (thermal challenge). PSII activity in these samples was assessed in triplicate using a Clarke-type electrode, set at 38°C. Data expressed as a percentage of the PSII activity remaining following thermal challenge, where 100% was that activity remaining in the growth temperature controls (*n* = 2).
(6.37 MB TIF)
