# Supplementary figures and images for: Identification of Components Associated with Thermal Acclimation of Photosystem II in Synechocystis sp. PCC6803
Source: PLoS One. 2010 May 6;5(5):e10511. doi: 10.1371/journal.pone.0010511 (PMC2865547; doi:10.1371/journal.pone.0010511)

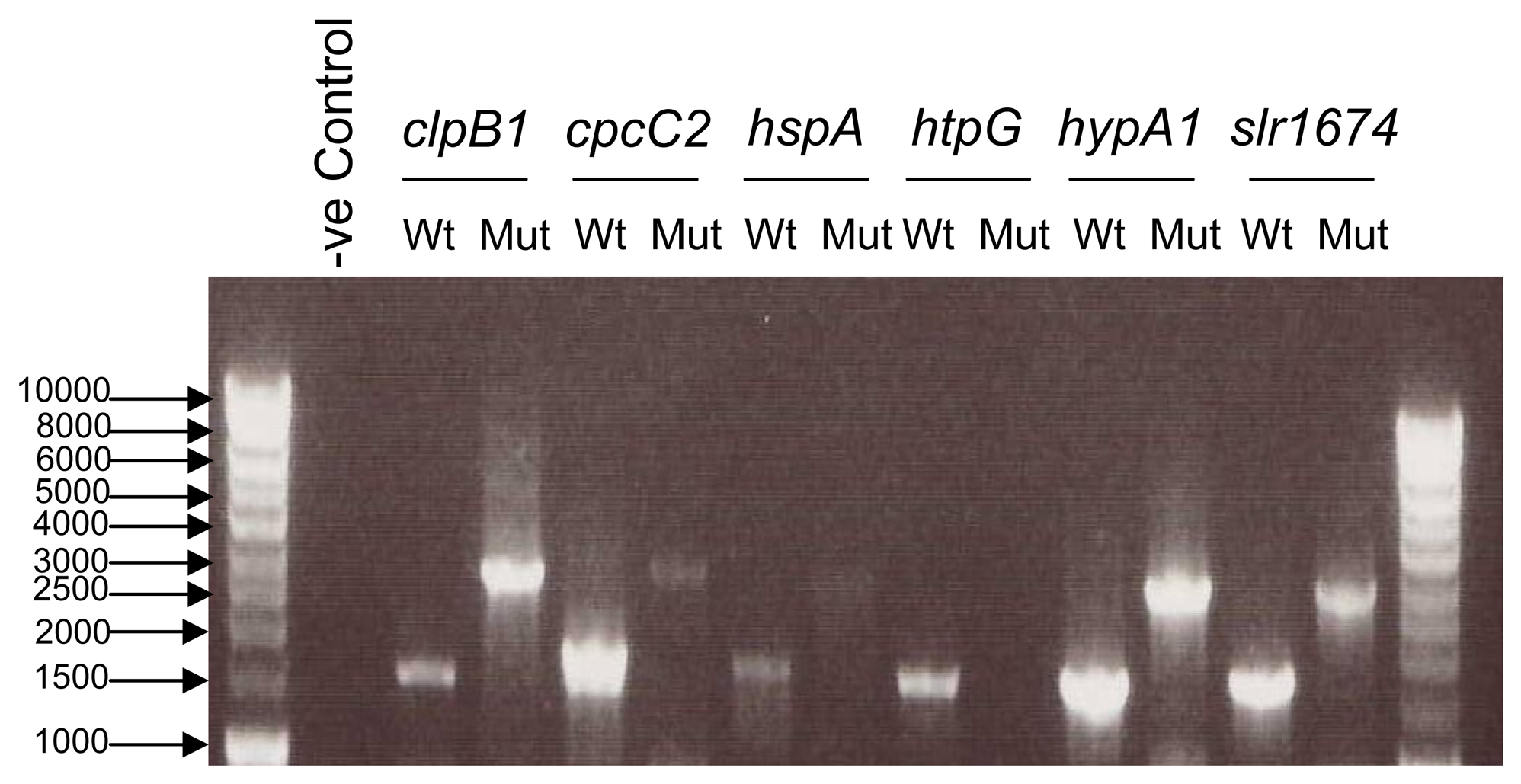

Supplement: Figure S1 — Confirmation of complete segregation of mutants. PCR was performed on wild-type (Wt) and potential mutant (Mut) genomic DNA using the indicated gene-specific primers. Expected amplicon sizes from Wt DNA were 1.6 to 1.8 kb. Complete segregation is indicated by the absence of a product in the Mut lanes at the Wt band size. (6.18 MB TIF) [file pone.0010511.s003.tif]

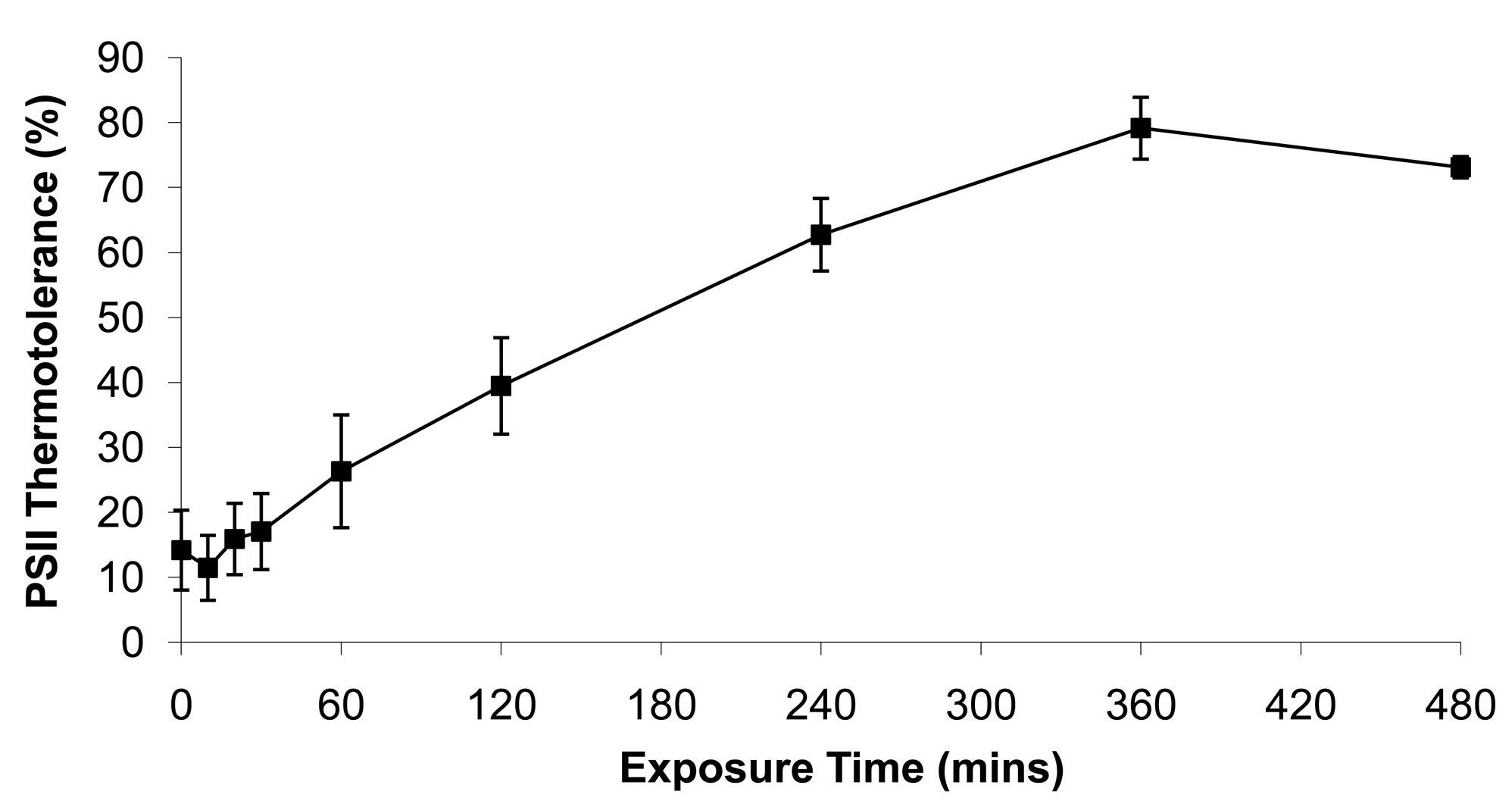

Supplement: Figure S2 — Acclimation of PSII measured at 38°C. Duplicate Synechocystis wild-type cultures were grown at 25°C to A730 = 1. The cultures were then shifted to 38°C and sampled over a time-course of 0 to 480 minutes. Samples were then treated for 1 hour in the dark at the growth temperature or at 46°C (thermal challenge). PSII activity in these samples was assessed in triplicate using a Clarke-type electrode, set at 38°C. Data expressed as a percentage of the PSII activity remaining following thermal challenge, where 100% was that activity remaining in the growth temperature controls (n = 2). (6.37 MB TIF) [file pone.0010511.s004.tif]
